# METABOLOMIC CHARACTERIZATION OF UNINTENTIONAL WEIGHT LOSS AMONG COMMUNITY-DWELLING OLDER ADULTS

**DOI:** 10.1093/geroni/igae098.3466

**Published:** 2024-12-31

**Authors:** Shanshan Yao, Megan Marron, Samaneh Farsijani, Ravi Shah, Venkatesh Murthy, Anne Newman

**Affiliations:** University of Pittsburgh, Pittsburgh, Pennsylvania, United States; University of Pitssburgh, Pittsburgh, Pennsylvania, United States; University of Pittsburgh, Pittsburgh, Pennsylvania, United States; Vanderbilt University Medical Center, Nashville, Tennessee, United States; University of Michgan, Ann Arbor, Michigan, United States; University of Pittsburgh, University of Pittsburgh, Pennsylvania, United States

## Abstract

Unintentional weight loss is associated with higher mortality and impaired mobility in older adults. Metabolomics can provide insight into yet unknown pathways of unintentional weight loss and inform its early prediction and targets for intervention. Here we investigated plasma metabolite associations of subsequent unintentional weight loss over 2 years (mean annual change [SD]=-1.5 [2.0] kg) in 2330 participants (mean age 74.6 years, 51% women, 37% Black) from the Health, Aging and Body Composition study. Metabolites were measured by liquid chromatography-mass spectrometry. Multinomial logistic regression models were used to identify the associations of each of the 478 metabolites with weight loss with/without an intention and weight gain (>3% annual weight gain/loss), relative to weight stability. The metabolite associations of unintentional weight loss differed from those of intentional weight loss and weight gain. Forty-seven metabolites were only significantly associated with unintentional weight loss after age, sex, race, current obesity, and multiple comparison adjustments compared with weight stable (FDR < 0.05). Lower levels of branched-chain amino acids, aromatic amino acids, phospholipids, long-chain poly-unsaturated triglycerides, and higher levels of amino acid derivatives, poly-unsaturated fatty acids, and carbohydrates were associated with higher odds of unintentional weight loss relative to weight stable. The identified metabolite associations were independent of smoking, energy expenditure, diet, inflammation, chronic conditions, and medication (< 20% attenuation). Altered amino acid metabolism, impaired mitochondrial fatty acid oxidation, and inflammaging implicated by the identified metabolites, which have been linked to insulin resistance and sarcopenia in literature, may precede unintentional weight loss in older adults.

